# TRPV1-linked neuropeptide axes in periodontitis and peri-implantitis

**DOI:** 10.3389/fendo.2026.1825849

**Published:** 2026-04-29

**Authors:** Yunzhi Lin, Qingfei He, Jianzhao Ni, Songting Ye, Ling Chen, Yun Wu, Chaofan Zhang

**Affiliations:** 1 Department of Stomatology, the First Affiliated Hospital, Fujian Medical University, Fuzhou, China; 2 Department of Stomatology, National Regional Medical Center, The First Affiliated Hospital, Fujian Medical University, Fuzhou, China; 3 Department of Stomatology, Zhouning General Hospital, Ningde, China; 4 Department of Orthopaedic Surgery, the First Affiliated Hospital, Fujian Medical University, Fuzhou, China; 5 Department of Orthopaedic Surgery, National Regional Medical Center, Binhai Campus of the First Affiliated Hospital, Fujian Medical University, Fuzhou, China; 6 Fujian Provincial Institute of Orthopedics, the First Affiliated Hospital, Fujian Medical University, Fuzhou, China

**Keywords:** calcitonin gene-related peptide, macrophage polarization, neuro-osteo-immune crosstalk, peri-implantitis, substance P, TRPV1

## Abstract

Periodontitis and peri-implantitis are the two most prevalent destructive diseases encountered in clinical dentistry, both of which arise from complex interactions among microbial challenge, host immune responses, and bone metabolism. Although these conditions share similar clinical manifestations, accumulating evidence indicates that the peripheral nervous system plays fundamentally distinct roles in their pathogenesis. In this narrative review, a structured literature search was conducted across multiple databases to synthesize and weigh current evidence on neuro-osteo-immune regulation in oral tissues. This review summarizes the bidirectional regulatory functions of neuropeptide axes, particularly the transient receptor potential vanilloid 1-calcitonin gene-related peptide (TRPV1-CGRP) axis and substance P-neurokinin 1 receptor (SP-NK1R) axis, within the bone-immune microenvironment. Current evidence suggests that CGRP is frequently associated with protective effects, including the promotion of osteogenesis and M2 macrophage polarization through the cAMP/PKA/CREB signaling cascade, whereas SP is more commonly associated with neurogenic inflammation and osteoclast activation in inflammatory settings, although both axes exhibit context-dependent effects. Special emphasis is placed on the impaired neural innervation of peri-implant tissues resulting from the absence of the periodontal ligament, as well as on the disruptive effects of titanium particle-induced foreign body reactions on neuro-immune crosstalk. By comparing the structural differences in neuro-osteo-immune regulation between these two diseases, we propose that periodontitis retains a certain capacity for self-regulation through periodontal ligament-mediated neural feedback, whereas peri-implantitis may be more prone to progressing toward chronic inflammation and bone destruction, potentially because of reduced neural input and persistent foreign body-related responses; however, this interpretation remains a conceptual model that requires direct validation in human disease. Finally, we discuss stage-specific therapeutic strategies targeting neuropeptide receptors and the prospective application of biomimetic implant coatings with neuromodulatory properties, aiming to provide a theoretical framework for future precision interventions in oral inflammatory diseases based on the neuro-osteo-immune axis.

## 1 Introduction

### 1.1 Periodontitis: a periodontal ligament-centered destructive inflammatory disease

Periodontitis is defined as a chronic destructive inflammatory disease affecting the tooth-supporting tissues. Initiated by dental plaque biofilms, it leads to progressive loss of periodontal attachment and alveolar bone resorption in susceptible individuals, ultimately resulting in tooth mobility and tooth loss. According to the most recent Global Burden of Disease (GBD) analysis, the prevalence of severe periodontitis is estimated to be approximately 11% of the adult population worldwide, corresponding to nearly 800 million affected individuals, with no substantial reduction in the overall disease burden over the past three decades (1). Periodontitis has been repeatedly demonstrated to exhibit bidirectional associations with multiple systemic conditions, including cardiovascular diseases, type 2 diabetes mellitus, and adverse pregnancy outcomes, thereby elevating it from a localized oral disorder to a chronic inflammatory condition with systemic health implications (2).

At the histological level, natural teeth are anchored to the alveolar bone via the periodontal ligament (PDL). The PDL is richly vascularized and densely innervated by sensory nerve fibers, serving as a critical interface for mechanotransduction, immune surveillance, and bone remodeling. The classical receptor activator of nuclear factor-κB ligand (RANKL)/osteoprotegerin (OPG) axis proposed in early osteoimmunology provided a mechanistic framework for inflammation-induced bone resorption. Subsequently, accumulating evidence has indicated that sensory nerve endings within the PDL not only transmit nociceptive signals but also actively regulate local immune responses and bone metabolism through the release of neuropeptides such as calcitonin gene-related peptide (CGRP) and substance P (SP). These findings have expanded the conceptual framework toward a more integrated and dynamic regulatory network encompassing the nervous, skeletal, and immune systems, referred to as the “neuro-osteo-immune” axis (3).

### 1.2 Peri-implantitis: a “periodontitis-like” disease lacking the periodontal ligament scaffold

Peri-implantitis is defined as an inflammatory disease affecting the tissues surrounding osseointegrated dental implants, characterized by inflammatory changes in the peri-implant mucosa accompanied by progressive loss of supporting peri-implant bone. Similar to periodontitis, dental plaque biofilms remain the primary initiating factor, and shared risk factors include smoking, diabetes mellitus, and poor oral hygiene. However, a fundamental distinction between the two conditions lies in their tissue architecture: unlike natural teeth, dental implants lack a periodontal ligament and instead rely on direct osseointegration for load transmission. Consequently, peri-implant tissues differ intrinsically from periodontal tissues in terms of neural innervation, vascular organization, and mechanosensory function (4).

Recent systematic reviews indicate that the prevalence of peri-implantitis is approximately 15-20% at the patient level and 10-13% at the implant level, with some studies reporting upper estimates exceeding 20% (5). With the continuous increase in the number of dental implants placed worldwide, the absolute number of peri-implantitis cases is steadily rising, even if relative prevalence rates remain stable. Similar to periodontitis, once peri-implantitis progresses to moderate or advanced stages, treatment often requires complex and costly surgical interventions, and clinical outcomes remain unpredictable.

### 1.3 From a binary to a tridimensional framework: bottlenecks in the prevention and management of periodontal diseases and the emerging role of the peripheral nervous system

From a public health perspective, neither periodontitis nor peri-implantitis is close to being effectively controlled or eliminated. Global Burden of Disease (GBD) analyses indicate that, from 1990 to 2021, the overall incidence and disability-adjusted life years attributable to periodontal diseases have remained largely unchanged worldwide, suggesting that current preventive and therapeutic strategies have failed to substantially reduce the global disease burden (2). In parallel, peri-implantitis has gradually emerged as a form of “hidden epidemic” accompanying the widespread adoption of implant therapy. Moreover, the lack of fully standardized diagnostic criteria implies that its true prevalence is likely underestimated (6).

Against this backdrop, a purely binary perspective centered on “bacteria-host immunity” is increasingly insufficient to account for certain clinical observations, such as cases showing continuous disease progression despite apparently adequate plaque control, or markedly divergent prognoses among different implants within the same oral cavity. These inconsistencies have prompted growing interest in a third regulatory dimension shaping the bone-immune microenvironment, namely the peripheral nervous system.

### 1.4 A new research paradigm from a neuro-osteo-immune perspective

In recent years, both basic and translational studies have revealed that nociceptive neurons distributed within bone and periodontal tissues, predominantly TRPV1^+^ and Piezo2^+^ sensory neurons, are capable of directly sensing mechanical stimuli, temperature changes, and bacterial products. Through the release of neuropeptides such as CGRP and substance P, these neurons exert profound effects on local immune cells, endothelial cells, and bone cells (3, 4, 7–9). A series of studies from the group led by Chiu IM further demonstrated that nociceptive neurons in the skin, lung, and gut can suppress neutrophil- and γδ T cell-mediated antimicrobial and inflammatory responses via TRPV1-dependent neuropeptide signaling, while, in specific contexts, they can also induce protective IL-17 responses and promote the activation of regulatory T cells (Tregs) (10, 11). Studies in non-oral tissues, including skin, lung, and gut, have demonstrated that nociceptive neurons can regulate immune responses in a context-dependent manner. These findings suggest that similar mechanisms may exist in oral tissues, although direct evidence remains limited.

Oral tissues are richly innervated by sensory nerves, and trigeminal nerve endings function not merely as passive nociceptors but as active effector units that modulate immune responses and bone metabolism through the release of neuropeptides, including CGRP and substance P (3, 10). This emerging neuro-osteo-immune perspective suggests that periodontitis and peri-implantitis may represent distinct clinical phenotypes shaped by dysregulation of the neuro-osteo-immune network, rather than being solely explained by bone-immune interactions. These observations suggest that periodontitis and peri-implantitis may be shaped by distinct neuro-osteo-immune contexts, an issue that is examined comparatively in later sections of this review.

Based on these considerations, this review focuses on the TRPV1-CGRP and SP-NK1R neuropeptide axes, systematically comparing their differential roles in periodontitis and peri-implantitis. By adopting a neuro-osteo-immune perspective, we aim to propose a conceptual framework and highlight potential therapeutic directions for these two oral inflammatory diseases that remain inadequately controlled in clinical practice.

In this review, we explicitly distinguish between established evidence and hypothesis-driven interpretations to avoid overextension of current findings. Importantly, much of the current evidence relevant to peri-implantitis remains preclinical or indirect, and direct validation in human peri-implant disease is still very limited.

## 2 Methods

### 2.1 Search strategy

This review was conducted as a narrative review informed by a structured literature search to identify relevant studies on TRPV1-linked neuropeptide signaling in bone-immune interactions, with a focus on periodontitis and peri-implantitis. Electronic databases including PubMed, Web of Science, and Scopus were searched for articles published between 2005 and 2025.

The search strategy combined keywords related to neural regulation and oral inflammatory bone diseases, including “TRPV1”, “CGRP”, “substance P”, “NK1R”, “neurogenic inflammation”, “osteoclast”, “macrophage polarization”, “angiogenesis”, “periodontitis”, and “peri-implantitis”.

### 2.2 Inclusion and exclusion criteria

Studies were included if they (1) investigated TRPV1 or neuropeptide signaling in immune or bone-related contexts (2), provided mechanistic insights into neuro-immune or neuro-osteogenic interactions, or (3) were directly related to oral inflammatory diseases. Both *in vitro*, *in vivo*, and clinical studies were considered.

Studies were excluded if they lacked mechanistic relevance, focused solely on unrelated systems, or were not available in English.

Given the limited number of studies directly addressing TRPV1 signaling in oral diseases, evidence from related systems (e.g., bone biology, neuroimmunology, and inflammatory models) was also incorporated. These studies were interpreted cautiously and used primarily to support mechanistic hypotheses rather than direct clinical conclusions. The identified literature was synthesized narratively, with direct evidence from oral diseases prioritized over indirect evidence from related non-oral systems.

## 3 Molecular regulatory mechanisms of core neuropeptide axes

### 3.1 The dual role of the TRPV1-CGRP axis in the bone-immune microenvironment: promotion of osteogenesis and suppression of inflammation

Transient receptor potential vanilloid 1 (TRPV1) is a non-selective cation channel predominantly expressed in small- to medium-diameter sensory neurons, classically responsible for sensing noxious heat, acidic microenvironments, and vanilloid compounds. Upon TRPV1 activation, calcium influx occurs at nerve terminals, leading to membrane depolarization and triggering the release of neuropeptides such as CGRP from synaptic vesicles into surrounding tissues. CGRP binds to its receptor complex composed of the calcitonin receptor-like receptor (CLR) and receptor activity-modifying protein 1 (RAMP1), thereby activating downstream signaling cascades including cyclic adenosine monophosphate/protein kinase A/cAMP response element-binding protein (cAMP/PKA/CREB) to regulate bone metabolism and immune responses (4, 12–14).

#### 3.1.1 Inhibition of osteoclastogenesis and promotion of osteogenesis and angiogenesis

At the level of bone metabolism, the TRPV1-CGRP axis is primarily characterized by suppression of osteoclast activity and concurrent promotion of osteogenesis and angiogenesis. Takahashi et al. demonstrated in an experimental periodontitis model that activation of TRPV1-expressing sensory nerves within periodontal tissues upregulated local CGRP levels, leading to a marked inhibition of osteoclast formation and alveolar bone resorption. In contrast, pharmacological blockade of TRPV1 or disruption of CGRP signaling exacerbated bone destruction. These findings suggest that the TRPV1-CGRP pathway may function as an endogenous protective mechanism for maintaining alveolar bone homeostasis (3).

In models of bone defect repair and implant osseointegration, the TRPV1-CGRP axis similarly exhibits pronounced pro-osteogenic and pro-angiogenic effects. Using neural interventions and genetic manipulation, Jiang et al. demonstrated that activation of TRPV1 in the trigeminal ganglion enhances CGRP release within bone defect sites and peri-implant tissues, thereby promoting osteogenic differentiation of bone marrow-derived mesenchymal stem cells as well as proliferation and migration of vascular endothelial cells (12, 15). These effects collectively accelerate bone bridging and neovascularization. Consistent with these findings, a growing body of studies on CGRP-mediated bone repair has shown that CGRP facilitates osteogenesis by upregulating key osteogenic transcription factors such as Runx2 and Osterix, suppresses RANKL-induced osteoclastogenesis, and enhances angiogenesis during fracture healing and peri-implant bone regeneration. Together, these observations support the notion that CGRP as a critical neuropeptide “growth factor” in the orchestration of bone remodeling and repair (16).

More recent studies have further refined the “CGRP-vasculature-osteogenesis” cascade into a testable molecular circuit. Specifically, CGRP can promote angiogenesis via endothelial focal adhesion kinase-protein kinase B-vascular endothelial growth factor (FAK-AKT-VEGF) signaling and stimulate bone vascular endothelial cells to secrete angiopoietin-like 4 (ANGPTL4), which in turn drives osteogenic differentiation of bone marrow mesenchymal stem cells (BMSCs) while suppressing adipogenic commitment, thereby accelerating bone defect repair *in vivo (*17). In parallel, potentiation of CGRP receptor (CLR) signaling has been shown to enhance the osteogenic capacity of BMSCs through the cAMP/PKA/CREB/JUNB pathway, while concomitantly promoting the formation of H-type vessels (CD31^hiEMCN^hi) and CGRP^+^ sensory nerve fibers (18). These findings may represent an amplifying “osteogenesis-angiogenesis-innervation” coupling loop, ultimately translating into increased bone mass and improved functional outcomes.

Notably, CGRP release from periosteal sensory nerves is not mediated exclusively by TRPV1. In an osseointegration model using high-elastic modulus implants, our group found that stress shielding leads to insufficient activation of mechanosensitive peri-bone neurons-predominantly Piezo2^+^ nociceptors-resulting in reduced local CGRP release. This reduction was accompanied by attenuation of focal adhesion kinase (FAK) signaling and downstream osteogenic programs in osteocytes, ultimately manifesting as delayed osseointegration. Conversely, exogenous CGRP supplementation or enhancement of the activity of these sensory neurons restored bone mass and vascularization at the bone-implant interface. Although this work primarily focused on Piezo2 rather than TRPV1 per se, both pathways converge on CGRP as a shared downstream effector, functionally highlighting a common “sensory nerve-CGRP-bone/vascular microenvironment” axis. In other words, the TRPV1-CGRP axis can be viewed as a key branch within a broader CGRP-centered sensory nerve-bone regulatory network, acting in concert with the mechanosensitive Piezo2-CGRP pathway to determine the quality and kinetics of peri-implant osseointegration (19).

Within this framework, the regulatory effects of the TRPV1-CGRP axis on osteoclastogenesis, osteogenesis, and angiogenesis should be interpreted not as outcomes of an isolated pathway, but as integral components of a broader CGRP-driven neuro-bone-vascular network. This systems-level view also provides a rationale for understanding how this axis interfaces with immune and infection-related processes within the local microenvironment.

#### 3.1.2 Immunomodulation and macrophage polarization

At the immunological level, the TRPV1-CGRP axis influences not only bone cells but also shapes the bone-immune microenvironment by regulating immune cells, particularly macrophages, thereby biasing the tissue milieu toward either a regenerative or a destructive state. Multiple *in vitro* and *in vivo* studies have shown that CGRP preferentially drives macrophage polarization from an M0/M1 phenotype toward an anti-inflammatory, pro-reparative M2 phenotype. In the absence of CGRP, macrophages are more likely to adopt an M1-like profile, characterized by elevated production of pro-inflammatory cytokines such as TNF-α and IL-1β, concomitant with impaired bone repair. In contrast, CGRP supplementation increases the proportion of M2 macrophages, upregulates canonical markers including Arg1 and CD206, and promotes bone regeneration and implant osseointegration (20).

Jiang et al. further demonstrated in an implant osseointegration model that activation of sensory neuron TRPV1 establishes an immunological milieu conducive to bone integration through a “TRPV1 → CGRP → macrophage” pathway. Inhibition of TRPV1 resulted in increased accumulation of M1 macrophages and a concomitant reduction in M2 macrophages around implants, leading to markedly impaired osseointegration. In contrast, pharmacological activation of TRPV1 or local supplementation with CGRP promoted macrophage polarization toward an M2 phenotype, reduced pro-inflammatory cytokine levels, and significantly improved bone-implant contact and bone volume fraction (4).

Broader reviews have also highlighted that CGRP suppresses Th1-type cytokine production and excessive leukocyte proliferation, thereby facilitating inflammation resolution and tissue remodeling during the later phases of bone repair (16). Taken together, these findings support the notion that, under conditions of “physiological repair,” the TRPV1-CGRP axis may establish a relatively restrained, M2-biased immune milieu by modulating macrophage polarization and limiting excessive inflammation, thereby creating permissive conditions for osteogenesis and angiogenesis.

Recent studies have further expanded the hierarchical role of the TRPV1-CGRP axis in bone-immune regulation. Qi et al. reported that during bone injury repair, TRPV1^+^ sensory neurons contribute to immune homeostasis not solely by modulating macrophage phenotypes, but also by releasing CGRP to act directly on osteoblasts. This signaling activates autophagy and promotes the production of the pro-resolving lipid mediator lipoxin A4 (LXA4), thereby suppressing neutrophil-associated inflammatory responses and facilitating the transition of inflammation toward the resolution phase. Conversely, disruption of TRPV1-CGRP signaling impairs this autophagy-LXA4 axis, leading to sustained inflammation accompanied by compromised bone repair (21).

#### 3.1.3 Roles in infectious diseases

In the context of infectious diseases, the function of the TRPV1-CGRP axis becomes more complex, exhibiting a dual nature characterized by both protective regulation and potential suppression of host defense.

Chiu IM and colleagues have demonstrated that nociceptive neurons, including TRPV1^+^ sensory neurons, constitute an integral component of the innate immune system. These neurons are capable of directly sensing bacterial toxins and pathogen-associated molecular patterns (PAMPs), and subsequently modulate the recruitment and effector functions of neutrophils, dendritic cells, and T cells through the release of neuropeptides such as CGRP, thereby influencing the overall trajectory of infection (11, 22).

In certain viral or bacterial infections, activation of TRPV1^+^ sensory neurons has been shown to confer protective effects. For instance, TRPV1^+^ fibers in the lung or vagal nerve can enhance host survival by modulating the intensity of local inflammation and maintaining barrier integrity. Conversely, genetic ablation or functional inhibition of these neurons leads to attenuated adaptive immune responses and a greater propensity for uncontrolled infection (23).

On the other hand, pathogens can also exploit the TRPV1-CGRP axis to undermine host defense mechanisms. In a Staphylococcus aureus-induced septic arthritis model, our group found that bacterial α-hemolysin directly activates TRPV1^+^ nociceptive neurons in the synovium, triggering robust CGRP release. The released CGRP acts on RAMP1 receptors expressed by CX3CR1^+^ synovial resident macrophages, leading to downregulation of chemokine and bactericidal gene expression. This suppresses immune cell recruitment and antimicrobial activity, thereby enabling persistent bacterial survival within the joint cavity and promoting long-term bone destruction (24). Consistently, Pinho-Ribeiro et al. observed a similar phenomenon in a cutaneous infection model, in which neuron-derived CGRP inhibited neutrophil recruitment and bactericidal function, whereas blockade of neural signaling or CGRP receptors enhanced immune clearance and improved infection outcomes (25).

Importantly, in the context of implant-associated infections, the timing of neural modulation appears to be critical. Our group has shown that optogenetic activation of Nav1.8^+^ nociceptive neurons prior to infection can induce a state of local preconditioned immunity via the CGRP-RAMP1/CLR axis, enhancing the competitive adhesion of host cells on implant surfaces and thereby effectively suppressing Staphylococcus aureus colonization and infection. In contrast, activation of these neurons after infection establishment failed to confer significant protection (26). These findings further indicate that the TRPV1-CGRP axis may contribute to immune priming and barrier reinforcement during the early phase of infection, whereas persistent activation after pathogen establishment could be exploited to dampen immune clearance.

Collectively, these studies indicate that the TRPV1-CGRP axis does not operate as a unidirectional regulator during infection, but rather dynamically shapes immune responses according to the stage of infection, pathogen characteristics, and tissue context. Early or appropriately tuned neural activation may enhance host defense, whereas excessive or sustained CGRP release during established or chronic infection can suppress immune clearance and favor persistence of the pathogen.

However, most of the available evidence derives from non-oral infection models, and whether a similar stage-dependent pattern operates in periodontitis or peri-implantitis remains to be directly established.

#### 3.1.4 Roles in oral diseases: periodontitis and peri-implantitis

In the oral cavity, available evidence suggests that the TRPV1-CGRP axis has context-dependent effects in periodontitis and peri-implantitis, although the strength and consistency of these effects appear to vary across experimental systems.

In experimental periodontitis models, Takahashi et al. used ligature-induced periodontal inflammation combined with neuropharmacological interventions and demonstrated that activation of TRPV1-expressing periodontal nerves increased local CGRP levels, inhibited osteoclastogenesis, and alleviated alveolar bone resorption. In contrast, genetic deletion or pharmacological inhibition of TRPV1 markedly aggravated periodontitis-associated bone loss (3). More recent studies have further indicated that CGRP-positive nerve fibers may exhibit a compensatory increase during the early stage of periodontitis, participating in the regulation of periodontal inflammatory intensity and bone remodeling (27).

However, not all oral disease findings support a uniformly protective interpretation of CGRP-related signaling. In the experimental study by Siddiqui et al., deletion of Tac1 significantly reduced ligature-induced periodontal bone loss and inflammation, whereas deletion of Calca produced only a marginal effect (28). This contrast suggests that the relative contribution of CGRP to periodontal pathology may be more variable and model-dependent than a simple uniformly protective framework would imply. Taken together, current oral evidence may be more consistent with a context-dependent and quantitatively asymmetric model, in which SP-associated inflammatory amplification is more consistently demonstrable, whereas CGRP-related protection may depend on disease stage, microbial burden, tissue compartment, and experimental readout.

In implant-related studies, the TRPV1-CGRP axis has been shown to influence both early osseointegration and the maintenance of immune homeostasis in peri-implant tissues. Activation of sensory neuron TRPV1 and subsequent CGRP release can enhance peri-implant bone defect repair, promote osteogenesis and angiogenesis, and create a regenerative immune milieu favorable for osseointegration by inducing M2 macrophage polarization and restraining excessive inflammation (4). Because direct evidence from oral disease models is still limited, the following interpretation should be considered hypothesis-generating rather than conclusively established. In light of findings from infection models such as septic arthritis (24), it is plausible that, under the chronic biofilm-driven inflammatory conditions characteristic of peri-implantitis, prolonged activation of the TRPV1-CGRP axis may conversely facilitate persistence of microbial biofilms. In this scenario, CGRP could suppress the bactericidal functions of key effector immune cells, including neutrophils and macrophages, thereby establishing a localized “immune-privileged” niche that favors chronic infection.

Based on evidence from bone remodeling, macrophage polarization, and systemic infection models, we propose a stage-dependent model in which the TRPV1-CGRP axis may exert protective effects during early inflammation, while prolonged activation under chronic biofilm conditions could suppress antimicrobial defenses. This stage-dependent model should be interpreted as a working hypothesis that integrates findings from bone repair, immune regulation, and systemic infection studies, rather than as a mechanism directly proven in oral inflammatory disease.

This hypothesis may provide a potential explanation for the clinical observation that bone destruction in periodontitis and peri-implantitis can persist despite the frequent absence of overt, severe symptoms. If validated in oral disease models, this stage-dependent hypothesis may help inform future therapeutic strategies targeting the TRPV1-CGRP axis, particularly by highlighting the need to account for disease stage and microbial burden rather than adopting a simplistic strategy of either global activation or complete blockade.

### 3.2 The SP-NK1R axis in inflammatory bone disease: context-dependent regulation of inflammation and bone remodeling

Substance P (SP) is a tachykinin neuropeptide released primarily from small- and medium-diameter nociceptive nerve fibers. Its principal receptor, neurokinin 1 receptor (NK1R), is widely expressed on vascular endothelial cells, fibroblasts, macrophages, osteoclast precursors, and various immune cell populations. In contrast to the TRPV1-CGRP axis, which is more often associated with pro-osteogenic and anti-inflammatory effects, in inflammatory bone disease, the SP-NK1R axis is often associated with pro-inflammatory and pro-osteoclastic signaling; however, its biological effects remain context-dependent and may vary with disease stage, tissue environment, and experimental system.

#### 3.2.1 Roles in bone metabolism: promotion of osteoclastogenesis and context-dependent bidirectional regulation of osteogenesis

At the level of bone metabolism, the SP-NK1R axis is generally characterized by a predominant pro-osteoclastic effect, accompanied by context-dependent bidirectional regulation of osteogenesis. Its biological actions are not static, but are jointly determined by SP concentration, temporal dynamics, and the local inflammatory milieu.

*In vitro* studies performed under non-inflammatory or low-inflammatory conditions have shown that SP can promote the proliferation of bone marrow-derived mesenchymal stem cells and osteoblasts via NK1R signaling, and within certain concentration ranges enhances late-stage mineralization. However, in the same experimental settings, SP simultaneously activates classical osteoclast-related pathways such as NF-κB and MAPK, thereby augmenting the differentiation of osteoclast precursors and bone resorptive activity, exhibiting a clear dose- and time-dependent bidirectional effect (29). Consistently, mice deficient in SP or NK1R display alterations in bone mineral density and microarchitecture, supporting an important regulatory role of this signaling axis in physiological bone remodeling (30).

Animal studies have further revealed the stage-specific and “quality-regulating” characteristics of SP during osteogenesis. Hofman et al. demonstrated in a rat fracture model that early blockade of SP-NK1R signaling significantly suppressed the expression of osteogenesis-related genes and was associated with reduced biomechanical strength at later healing stages (6 weeks and 3 months post-fracture). Notably, callus volume remained largely unchanged, suggesting that SP is more likely to influence the maturation and mechanical quality of newly formed bone rather than simply determining bone mass (31).

However, within inflammatory or osteoclast-driven bone microenvironments, the functional profile of the SP-NK1R axis shifts markedly. A number of studies have shown that in the presence of RANKL or elevated inflammatory cytokines such as TNF-α and IL-1β, SP acts synergistically with these pro-inflammatory signals to increase the RANKL/OPG ratio and markedly enhance the activity of osteoclast precursors and mature osteoclasts, thereby accelerating bone resorption (32, 33). Recent models of inflammation-associated bone loss have further confirmed that blockade of NK1R effectively suppresses inflammation-driven osteoclast activation and mitigates bone destruction, supporting the view that SP tends to exert predominantly pro-resorptive effects under “inflammation-osteoclast” conditions (34).

Taken together, the role of the SP-NK1R axis in bone metabolism cannot be simply categorized as either pro-osteogenic or pro-osteoclastic, but is better understood as a highly microenvironment-dependent regulatory mechanism. During tissue repair or under low-inflammatory conditions, SP may participate in the qualitative regulation of osteogenesis, whereas in chronic inflammatory settings or RANKL-dominated contexts, its activity appears to shift toward enhanced osteoclastogenesis and accelerated bone resorption. These context-dependent findings provide a useful conceptual basis for interpreting the potential contribution of SP to inflammatory bone diseases, although direct evidence in periodontitis and peri-implantitis must be evaluated separately.

#### 3.2.2 Inflammatory amplification and neurogenic inflammation: pro-inflammatory and immuno-osteodestructive actions of SP

Substance P is a well-recognized mediator of neurogenic inflammation. Upon release, SP rapidly induces vasodilation and increases endothelial permeability, thereby creating permissive pathways for the migration and extravasation of immune cells such as neutrophils and monocytes/macrophages (35).

SP can also potentiate the responsiveness of immune and bone cells to pathogens or inflammatory stimuli such as bacteria and cytokines. For example, recent experimental studies have shown that under Staphylococcus aureus infection, SP-NK1R signaling amplifies the reactions of bone cells-including both osteoblasts and osteoclasts-to inflammatory and immunological challenges, enhancing the expression of inflammatory mediators and bone resorption-related factors, thereby exacerbating infection-associated bone destruction (32).

Accordingly, under conditions of chronic inflammation or infection, the SP-NK1R axis may act as an “inflammation-osteoclast amplification module” in experimental settings, promoting the transition from localized inflammatory stimuli to sustained bone-destructive responses. Whether this amplification pattern is fully recapitulated in oral inflammatory diseases remains to be directly established.

#### 3.2.3 The SP-NK1R axis in periodontitis: amplification of hypoxic and osteoclastic signals

Periodontal tissues are prone to local ischemia and hypoxia under chronic inflammation and plaque-induced compression. Studies have demonstrated that SP expression is markedly elevated in periodontitis, while NK1R is widely expressed in gingival vasculature and inflammatory cells (36). Fristad and colleagues systematically revealed through immunohistochemical analyses the broad distribution of SP receptors in periodontal tissues, including cementocytes, osteoblasts, vascular endothelial cells, and connective tissue cells, indicating that SP signaling may contribute importantly to both the maintenance of periodontal homeostasis and the orchestration of inflammatory responses (37).

Further mechanistic insights were provided by Azuma et al., who demonstrated that SP potentiates the inhibitory effects of Porphyromonas gingivalis lipopolysaccharide (P-LPS) on osteoblast differentiation via NK1R signaling. Specifically, SP markedly reduced the expression of bone sialoprotein (BSP), osteopontin (OPN), osteocalcin (OCN), and the osteogenic transcription factor Cbfa-1, thereby suppressing bone formation. These findings suggest that, within an inflammatory milieu, SP may interfere with osteogenesis through multiple convergent pathways (38).

In addition, the study by Winning et al. provided complementary insights from a stem cell perspective regarding the regulation of SP receptors. Under inflammatory conditions, TNF-α and IL-1β were shown to downregulate the expression of the SP receptor TACR1 in STRO-1^+^ periodontal ligament stem cells (PDLSCs), suggesting that inflammation may modulate SP signaling by reducing receptor availability. This observation is consistent with the pro-inflammatory role of SP in periodontitis (39).

Yan et al. further demonstrated in a periodontitis model that SP upregulates the expression of hypoxia-inducible factor-1 alpha (HIF-1α) in bone marrow-derived osteoclast precursors and gingival fibroblasts *in vitro*, while significantly increasing the RANKL/OPG ratio, thereby promoting osteoclastogenesis and alveolar bone resorption (40).

Further animal studies have suggested that when hypoxia coexists with LPS challenge, the pro-osteoclastic effects of SP are further amplified, supporting the view that SP may function as an important amplification node within a “hypoxia-inflammation-bone resorption” cascade (41). This notion is consistent with clinical features frequently observed in periodontitis, including gingival microvascular dilation, increased exudation, and progressive alveolar bone loss.

Taken together, current evidence suggests that in periodontitis, SP may contribute to bone homeostasis disruption not only by inhibiting osteoblast differentiation (38) and promoting inflammatory responses (39), but also by enhancing osteoclastogenic signaling in hypoxic environments through upregulation of HIF-1α and an increased RANKL/OPG ratio. These convergent observations suggest that SP may contribute importantly to inflammatory amplification and osteoclastogenic signaling in periodontitis; however, the magnitude and consistency of this effect may vary across experimental settings and require further confirmation in human disease (41).

#### 3.2.4 The SP-NK1R axis in peri-implant tissues: from nociception to immune dysregulation

The neural innervation of peri-implant soft and hard tissues differs fundamentally from that of natural teeth. Yamaza et al., using immunohistochemical analyses, identified SP- and NK1R-positive nerve fibers and vascular structures within the subepithelial connective tissue surrounding successfully osseointegrated implants. These findings suggest that regenerated nerve fibers not only participate in the perception of mechanical stimuli and pain, but may also contribute to neutrophil extravasation and the regulation of local inflammatory responses (42).

Although direct comparative studies on the role of the SP-NK1R axis in peri-implantitis versus periodontitis remain limited, available evidence suggests that SP- and NK1R-positive neural and vascular elements are present in peri-implant tissues. Based on these observations, it is plausible that, in peri-implant tissues, SP may rely more heavily on NK1R-positive cells within bone marrow and peri-implant soft tissues to exert its effects.

In the setting of foreign body reactions, this pathway may contribute to inflammatory amplification and bone resorption; however, direct mechanistic evidence in peri-implantitis remains insufficient. Thus, the proposed role of SP-NK1R signaling in peri-implantitis should currently be regarded as a conceptual model derived from limited structural and mechanistic evidence, rather than as a definitive disease mechanism.

#### 3.2.5 Systemic stress, psychological factors, and SP upregulation: projecting stress signals to bone and periodontal tissues

Beyond local stimuli, the SP-NK1R axis is also modulated by systemic neuroendocrine-immune status. Previous reviews have highlighted that the SP/NK1R pathway is implicated not only in pain and neuroinflammation, but also in stress responses, anxiety/depression, and chronic disease states (35). A recent study published in PNAS further demonstrated that systemic stressors such as sleep deprivation selectively increase SP release in periodontal tissues via trigeminal neural pathways. Excessive SP acting on NK1R disrupted local immune homeostasis and aggravated periodontal bone destruction, whereas pharmacological blockade of NK1R effectively reversed this process (43).

In the context of bone metabolism, studies have reported that a systemic decline in SP signaling-such as that associated with aging, neural injury, or alterations in the bone marrow microenvironment-is correlated with reduced bone mass and impaired bone repair capacity (44).

These findings suggest that the SP-NK1R axis may function as an important molecular link between systemic stress or metabolic states and local bone-periodontal responses. In the settings of periodontal tissue repair or destruction, it is plausible that this pathway contributes to the translation of systemic factors-such as psychological stress, metabolic dysregulation, and altered neural function-into local inflammatory dysregulation and imbalanced bone remodeling. However, the extent to which this systemic-to-local SP-NK1R axis operates in peri-implant disease remains to be directly clarified. To improve clarity, the principal similarities and differences between the TRPV1-CGRP and SP-NK1R axes across inflammation, osteoclastogenesis, osteogenesis/repair, disease context, and translational implications are summarized in **Table 1**.

**Table 1:** Comparison of the TRPV1-CGRP and SP-NK1R axes in neuro-osteo-immune regulation.

Feature	TRPV1-CGRP axis	SP-NK1R axis
Inflammation/immune tone	Often associated with inflammation restraint or resolution during tissue repair and early healing, but these effects are context-dependent and may vary with infection stage and tissue environment.	More often associated with pro-inflammatory signaling and neurogenic inflammatory amplification, especially under conditions of tissue injury, infection, hypoxia, or chronic stress.
Effects on osteoclastogenesis	Commonly suppresses osteoclastogenesis and bone resorption in periodontitis-related and bone repair models.	Commonly enhances osteoclastogenic signaling, particularly in inflammatory or hypoxic settings.
Effects on osteogenesis/repair	Promotes osteogenesis, angiogenesis, and reparative immune regulation in several bone repair and osseointegration models.	Shows context-dependent effects: under low-inflammatory conditions it may participate in aspects of osteogenic regulation, whereas under inflammatory conditions it tends to favor bone resorption over repair.
Role in periodontitis	Experimental studies support a protective effect in limiting osteoclastogenesis and bone loss, although the magnitude and consistency of this effect may vary across oral models.	More consistently associated with inflammatory amplification, hypoxia-related osteoclastogenic signaling, and progressive periodontal bone destruction.
Role in peri-implantitis	Proposed to support early osseointegration and reparative immune regulation; whether similar signaling remains beneficial in chronic biofilm-driven peri-implantitis remains to be directly established.	Likely contributes to neurogenic inflammatory amplification in peri-implant tissues, but this interpretation remains largely conceptual because direct mechanistic evidence in peri-implantitis is limited.
Translational implication	A potential target for stage-specific, local, time-restricted enhancement during regeneration or early osseointegration, but current support remains preclinical.	A potential target for local inhibition during active inflammatory and osteolytic phases, although current therapeutic support also remains largely preclinical.

## 4 Comparative neuro-osteo-immune dysregulation in periodontitis and peri-implantitis

### 4.1 Presence versus absence of the periodontal ligament: structural determinants of distinct neuromodulatory patterns

Although periodontitis and peri-implantitis share highly similar clinical manifestations and radiographic features, their fundamental differences in tissue architecture argue against a simple equivalence in neuro-osteo-immune regulation. Natural teeth are connected to the alveolar bone through the periodontal ligament (PDL), a specialized structure that not only provides mechanical buffering and load transmission but also serves as a highly vascularized and densely innervated biological interface. The PDL is enriched in mechanoreceptors and nociceptors, enabling rapid sensing of mechanical loading and the release of neurotrophic factors such as CGRP to maintain bone homeostasis (3, 4). Multiple histological and immunohistochemical studies have confirmed the dense distribution of CGRP^+^ and SP^+^ sensory nerve fibers within the PDL, with their density dynamically changing in response to mechanical stimulation and tissue remodeling status, supporting important roles in local bone metabolism and inflammatory regulation (45–47). Consistent with this concept, studies in experimental periodontitis have shown that TRPV1 activation can suppress osteoclastogenesis by upregulating CGRP release (3).

In contrast, dental implants connect to host bone through direct osseointegration, essentially forming a direct implant-bone interface that lacks the buffering layer and neurovascular network provided by the PDL. This structural simplification is likely to reduce local neural input and may blunt peri-bone sensing of mechanical and inflammatory cues, thereby potentially weakening local immunomodulatory capacity under microbial challenge. Studies in osseointegration models are consistent with this interpretation: genetic deletion or pharmacological blockade of TRPV1 increases the proportion of M1 macrophages and reduces M2 macrophages in peri-implant tissues, significantly impairing osseointegration. Conversely, TRPV1 activation or exogenous CGRP supplementation promotes bone-implant integration and improves the local immune microenvironment (4). However, whether this structurally linked reduction in neural regulation directly contributes to peri-implantitis remains to be established.

### 4.2 Neuropeptide imbalance: from “protective-destructive equilibrium” to a pro-inflammatory dominant state

Under healthy conditions or during mild inflammation, the TRPV1-CGRP and SP-NK1R axes in periodontal tissues may be viewed as maintaining a dynamic equilibrium. CGRP tends to inhibit osteoclastogenesis, promote osteogenic differentiation, and drive macrophage polarization toward a reparative phenotype, whereas SP mediates vasodilation, immune cell recruitment, and nociceptive signaling in response to tissue injury and acute inflammation.

During chronic periodontitis, this balance is gradually disrupted. Both animal models and human tissue studies have shown that SP expression remains persistently elevated within periodontal lesions, whereas the relative expression or bioactivity of CGRP becomes insufficient, suggesting a shift of the SP/CGRP ratio toward a more pro-inflammatory state. This neuropeptide imbalance correlates significantly with an increased RANKL/OPG ratio, higher numbers of osteoclasts, and greater extent of alveolar bone resorption (28, 40).

Notably, recent studies have also indicated that CGRP participates in periodontal tissue repair and regeneration under specific conditions, particularly through its roles in regulating osteogenic differentiation and matrix mineralization of periodontal ligament cells. These findings further underscore the dose- and time-dependent bidirectional nature of neuropeptide signaling within the periodontal microenvironment (27).

In peri-implantitis, this neuropeptide imbalance may be even more pronounced. Because baseline neural regulation is structurally reduced in peri-implant tissues, neuropeptide imbalance may be more pronounced in peri-implantitis than in periodontitis (48, 49). On the other hand, peri-implant tissues exhibit sensory nerve regeneration and expression of the SP-NK1R system within the mucosa, epithelium, and inflammatory cells, providing anatomical and molecular conditions that may favor amplification of neurogenic inflammation (42). Moreover, the mechanisms underlying marginal bone loss around osseointegrated implants have long been interpreted through two complementary frameworks-plaque-induced inflammation and foreign body/material-related stimulation (including particulate debris, corrosion products, and abnormal mechanical loading). Regardless of the conceptual model adopted, these external stimuli-biofilm burden, material/particle release, and mechanical perturbations-can activate the macrophage-osteoclast axis and promote bone resorption (50–52). Taken together, these observations support a conceptual model in which peri-implantitis may be associated with a neuro-immune microenvironment biased toward pro-inflammatory and pro-osteoclastic dominance. However, this model is based largely on preclinical and indirect evidence, and direct validation in human peri-implantitis remains limited.

### 4.3 Titanium particles and neuro-immune interactions

During long-term clinical service of dental implants, titanium particles (TiPs) of varying sizes may be released from implant surfaces as a result of functional loading, micromotion-induced friction, or mechanical debridement and maintenance procedures. Growing evidence suggests that these particles are not merely inert byproducts, but may contribute to and amplify inflammatory processes associated with peri-implantitis, and are increasingly considered an important factor affecting the long-term stability of implants (50). Although these findings provide a plausible framework for interpreting neuro-immune dysregulation in peri-implantitis, most of the available support remains indirect or preclinical, and the extent to which these mechanisms operate in human peri-implant disease has not yet been clearly established.

#### 4.3.1 Titanium particle-induced aberrant macrophage polarization and inflammasome activation

Micro- and nanoscale titanium particles can be phagocytosed by macrophages, subsequently triggering NOD-like receptor family pyrin domain containing 3 (NLRP3) inflammasome activation through mechanisms such as lysosomal damage and danger-associated signaling. This process leads to caspase-1 activation and promotes the maturation and release of IL-1β and IL-18, thereby amplifying local inflammatory responses (53–55).

During this process, titanium particles induce macrophages to produce pro-inflammatory cytokines such as TNF-α, IL-1β, and IL-6, driving the cells toward a predominantly M1-like inflammatory phenotype and thereby potentially contributing to a pro-inflammatory microenvironment in peri-implant tissues (56). Transcriptomic analyses of clinically relevant peri-implant gingival tissues have similarly revealed upregulation of inflammatory mediators-including IL-1β, IL-8, and IL-18-in samples associated with implant failure or inflammation, together with alterations in immune cell composition, supporting the view that titanium particles in immune dysregulation (51). Moreover, in particle-induced osteolysis models, particulate stimulation has been shown to enhance RANKL expression in stromal and fibroblastic cells via cyclooxygenase-2/prostaglandin E2 (COX-2/PGE2) signaling pathways, thereby facilitating osteoclastogenesis and promoting bone resorption (57).

#### 4.3.2 Amplifying interactions among titanium particles, inflammatory mediators, and sensory nerves

Within an inflammatory milieu, pro-inflammatory cytokines induced by titanium particles may also act on peri-implant sensory nerve fibers, including TRPV1^+^ nociceptive terminals, thereby potentially enhancing neuropeptide-related inflammatory responses. In implant-related contexts, such as placement procedures, maintenance with ultrasonic debridement, or implantoplasty, released titanium particles have been shown to stimulate macrophages and surrounding cells to produce TNF-α, IL-1β, IL-6, and other inflammatory mediators, which are associated with increased osteoclastogenesis and bone resorption (52, 56, 58, 59).

Subsequently, TNF-α has been shown to upregulate TRPV1 expression in dorsal root ganglion neurons and to potentiate TRPV1-associated CGRP release mechanisms (60, 61). Likewise, IL-1β can induce SP release from sensory neurons (62). Taken together, these observations suggest a plausible positive feedback loop in which inflammatory mediators activate sensory nerves and promote neuropeptide secretion, further amplifying local inflammation. However, whether this loop is directly operative in peri-implantitis remains to be established. In addition, under LPS-primed conditions, titanium ions can aggregate into particulate forms and trigger inflammasome-dependent IL-1β release, suggesting that a “particle/ion-inflammasome-IL-1β/IL-18” axis may also contribute to pro-inflammatory amplification around implants (63).

#### 4.3.3 Regulatory implications of titanium surface topography on the neuro-immune microenvironment

Beyond the particles themselves, the physicochemical properties of titanium implant surfaces-such as topography and wettability-can markedly influence macrophage activation states and their cytokine profiles. Numerous studies have shown that hydrophilic, micro-rough titanium surfaces (e.g., modSLA) tend to drive macrophages toward a more anti-inflammatory and reparative (M2-like) phenotype, characterized by reduced production of pro-inflammatory mediators such as TNF-α, IL-1β, and IL-6, and enhanced expression of reparative signals including IL-4 and IL-10. Moreover, conditioned media derived from macrophages cultured on such surfaces have been reported to promote osteogenic pathways and upregulate osteogenesis-related genes (64, 65).

In contrast, smoother and less hydrophilic (more hydrophobic) titanium surfaces are more prone to induce an M1-like inflammatory phenotype, manifested by elevated levels of IL-1β, IL-6, and TNF-α. Increasing surface wettability and roughness has been shown to shift macrophage responses toward a more anti-inflammatory state (64, 66). Further *in vivo* evidence supports the concept that such “surface-mediated immunomodulation” can be translated into differential bone healing outcomes. Ma et al. demonstrated in a rat model that nanotopographical titanium surfaces modulate macrophage polarization, reduce early inflammatory burden, and enhance bone-implant contact (BIC) and osseointegration quality, indicating that the macrophage-dominated early inflammatory phase may represent an important intermediary through which surface morphology influences bone healing (67). Under unfavorable systemic conditions such as type 2 diabetes, Lee et al. further showed that surface modification capable of “reconstructing” local macrophage homeostasis-characterized by reduced M1 and enhanced M2 phenotypes-significantly improved bone healing and osseointegration parameters, providing stronger *in vivo* support for a link between immune microenvironment optimization to enhanced osseointegration (68). Additionally, Abaricia et al. observed in both *in vitro* and *in vivo* experiments that different commercial implant surfaces induced markedly distinct macrophage secretory profiles and proportions of CD80^hi (M1-like) versus CD206^^+^ (M2-like) adherent cells, suggesting that the “surface-immune phenotype” relationship is not merely an *in vitro* artifact but emerges early *in vivo* (69). At the clinical level, hydrophilic-surface implants have demonstrated favorable short-term marginal bone levels and stability outcomes (70), and recent randomized controlled trials comparing hydrophilic and hydrophobic surfaces have further provided evidence for the clinical relevance of surface properties to marginal bone changes and implant stability during one-year follow-up (71).

Moreover, pro-inflammatory cytokines such as TNF-α and IL-1β can lower the excitation threshold of sensory nerve terminals and enhance the release of TRPV1/CGRP- and SP-related neuropeptides. These observations provide a plausible mechanistic basis for the idea that differences in “surface-induced inflammatory intensity” may be translated into distinct levels of neuro-immune interaction-for example, TNF-α upregulates TRPV1 expression and facilitates TRPV1-dependent CGRP release, while IL-1β can directly induce SP secretion (60–62). Accordingly, implant surface topography may be considered not only an important determinant of immunomodulation, but also a potential indirect modulator of the neuro-immune microenvironment through its influence on local cytokine profiles and downstream neuropeptide signaling.

**Figure 1:**
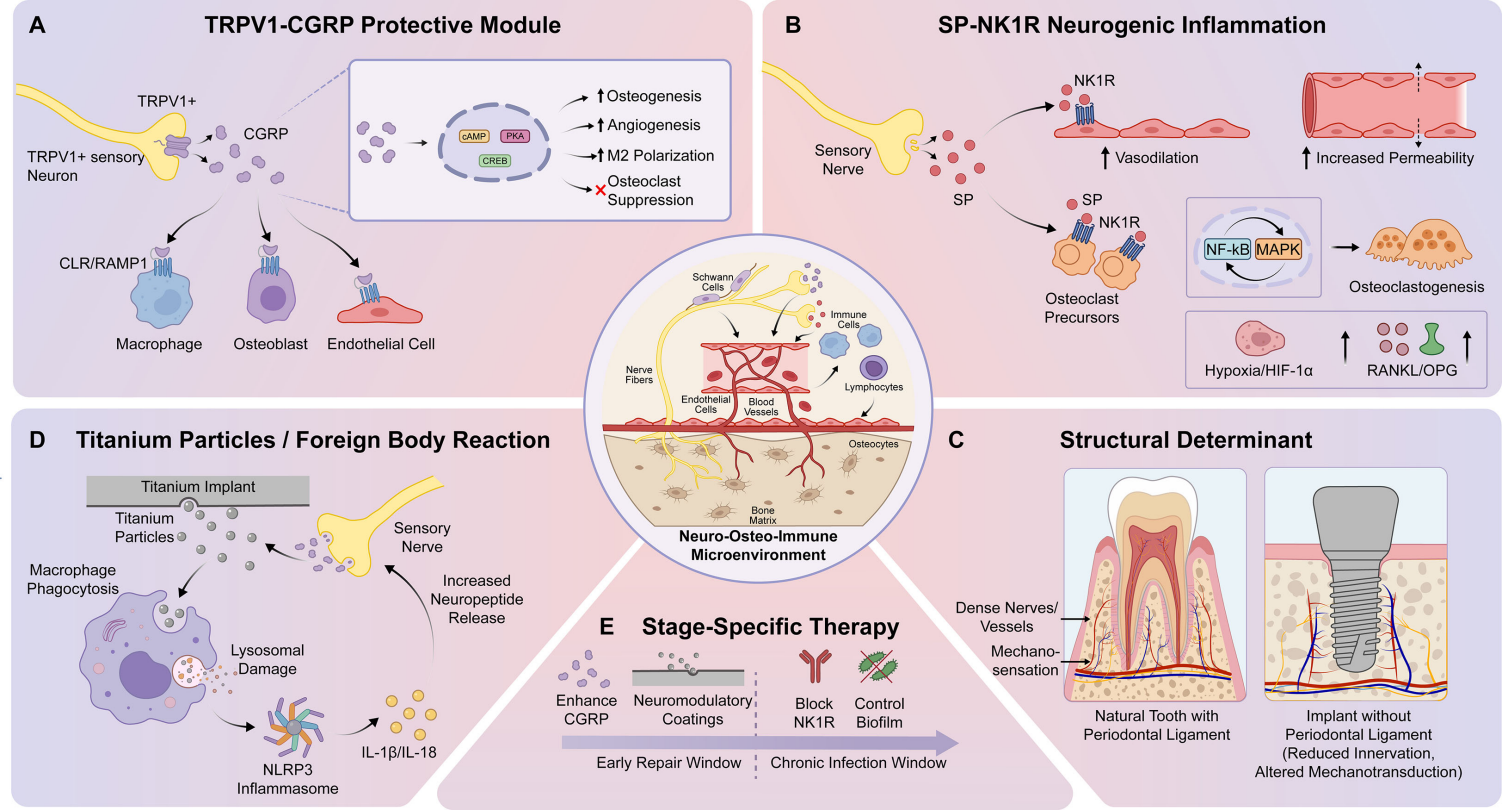
Neuro-osteo-immune framework in peri-implant diseases. Schematic illustration of neuro-osteo-immune regulation in periodontitis and peri-implantitis. **(A)** TRPV1-CGRP axis promotes osteogenesis, angiogenesis, and M2 macrophage polarization while suppressing osteoclastogenesis. **(B)** SP-NK1R signaling drives neurogenic inflammation, vasodilation, and RANKL-dependent bone resorption. **(C)** Structural determinant of the periodontal ligament versus implant interface highlighting differences in innervation and mechanosensation. **(D)** Titanium particle-induced foreign body reaction involving macrophage phagocytosis, NLRP3 inflammasome activation, and IL-1β/IL-18 release. **(E)** Stage-specific therapeutic strategy balancing enhancement of CGRP signaling during early repair and blockade of NK1R during chronic infection.

From this perspective, future implant design may benefit from considering not only biomechanical endpoints such as bone-implant contact (BIC), but also how surface properties shape the interaction between immune cells and sensory nerves. Such an integrative strategy could help inform approaches aimed at reducing chronic inflammation and neurogenically amplified bone resorption, although this possibility requires further validation. An overview of the neuro-osteo-immune regulatory framework in periodontitis and peri-implantitis is shown in **Figure 1**. To facilitate comparison across the major neuro-osteo-immune dimensions discussed above, the principal differences between periodontitis and peri-implantitis are summarized in **Table 2**.

**Table 2:** Compact comparison of neuro-osteo-immune features in periodontitis and peri-implantitis.

Dimension	Periodontitis	Peri-implantitis
Innervation	Dense PDL-associated sensory innervation with CGRP^+^/SP^+^ fibers; preserved neuropeptide-mediated regulation.	No PDL neural scaffold; compensatory osseoperception and regenerated peri-implant neural/vascular elements, but simplified neural regulation.
Vascularity	Highly vascularized PDL neurovascular interface supports surveillance and repair.	Altered peri-implant vascular organization without native PDL interface; more vulnerable to inflammation-driven dysregulation.
Mechanosensation	Preserved tooth-PDL mechano-neural feedback.	Loss of tooth-PDL feedback; reliance on altered osseoperception.
Foreign-body response	Absent; pathology mainly reflects biofilm-host dysregulation.	Present; titanium particles/corrosion/mechanical perturbation may amplify inflammation and osteoclastogenesis.
Likely therapeutic implications	Stage-specific neuropeptide modulation to support repair and limit chronic inflammatory amplification.	Combined control of biofilm and foreign-body-related inflammation; potential local neuropeptide modulation plus future neuromodulatory biomaterials (conceptual; requires direct validation in human disease).

## 5 Clinical translation: near-term opportunities and future biomaterial directions

### 5.1 Near-term opportunities: stage-specific modulation of neuropeptide axes

At present, these proposals should be regarded as hypothesis-informed translational directions rather than evidence-based clinical recommendations, because direct support from human intervention studies remains lacking. From a translational perspective, the most realistic near-term opportunities lie not in broad systemic neuromodulation, but in localized and time-restricted modulation of neuropeptide axes as an adjunct to established infection-control strategies. Current evidence suggests that the TRPV1-CGRP and SP-NK1R pathways should be considered within a stage-specific framework rather than as uniformly “beneficial” or “harmful” targets.

For the TRPV1-CGRP axis, the most plausible near-term application is short-duration local enhancement during defined windows of periodontal regeneration or early osseointegration, particularly after biofilm burden has been reduced and active infection is limited. In this setting, controlled augmentation of CGRP-related signaling may help improve macrophage polarization, early bone formation, and implant integration (3, 4, 20). However, this strategy should not be extrapolated indiscriminately to biofilm-dominated disease, because evidence from infection models indicates that sustained CGRP signaling can suppress antimicrobial effector functions and favor pathogen persistence (9, 24, 25). Accordingly, TRPV1-CGRP-targeted interventions appear more suitable as temporally restricted, locally delivered adjuncts than as continuously activated therapeutic approaches. At present, these proposals should be regarded as translationally relevant but still preclinical strategies, since direct support from human intervention studies in periodontitis or peri-implantitis remains lacking. At the same time, conflicting oral findings indicate that the contribution of CGRP-related signaling may not be uniformly protective across all periodontal settings, and future translational strategies should therefore account for model-dependent variability as well as disease stage.

By contrast, the SP-NK1R axis appears more consistently associated with inflammatory amplification and bone destruction. Experimental studies have shown that NK1R blockade can reduce inflammatory cytokine expression, inhibit osteoclast differentiation, and attenuate bone loss, including under stress-aggravated periodontal conditions (28, 40, 43). This makes local and controllable NK1R antagonism a comparatively more practical near-term strategy, especially during phases characterized by active inflammation, ongoing bone resorption, and pain sensitization. In clinical terms, such an approach would be more rational as an adjunct to conventional debridement, biofilm control, and, when indicated, antimicrobial therapy, rather than as a stand-alone intervention.

Taken together, the most immediately testable translational strategy is a stage-stratified approach: supporting TRPV1-CGRP-mediated reparative signaling during early healing and osseointegration, while avoiding sustained activation during established infection and considering inhibition of SP-NK1R-mediated neurogenic inflammatory amplification during chronic inflammatory phases. This framework may better reflect the dynamic pathology of periodontitis and peri-implantitis than long-term activation or suppression of any single neuropeptide pathway. Similarly, although local NK1R antagonism may represent a more testable translational direction, current support remains largely preclinical and insufficient for definitive clinical recommendation.

### 5.2 Future biomaterial directions: neuromodulatory implant design

A longer-term translational direction is the development of neuromodulatory implant materials that not only optimize osseointegration mechanically, but also shape the local neuro-immune microenvironment. Through approaches such as elastic modulus matching, micro-/nano-topographical surface engineering, and localized delivery of neurotrophic or neuropeptide-related cues, such materials may promote favorable nerve-immune-bone coupling around implants and partially reconstruct aspects of periodontal ligament-like sensory regulation (48, 72).

At present, however, this strategy remains largely preclinical. Existing support derives mainly from *in vitro* studies and small-animal models showing that manipulation of implant surface topography, stiffness, or neuromodulatory cues can promote nerve fiber ingrowth and improve osseointegration-related outcomes (4, 67, 68). Although these findings highlight the biological plausibility of “neuro-bone coupling” as a design principle, they do not yet establish clinical efficacy in the human oral environment.

Importantly, there is still a lack of long-term clinical evidence demonstrating that promotion of peri-implant neural ingrowth improves implant survival or reduces the risk of peri-implantitis. An additional unresolved issue is whether enhancement of local neural signaling could have adverse immunological consequences under chronic infectious conditions (50, 71). For these reasons, neuromodulatory implant design should currently be regarded as a future biomaterial strategy rather than an immediately deployable clinical solution.

## 6 Conclusions and perspectives

Overall, periodontitis and peri-implantitis can be regarded as two disease phenotypes that develop within distinct neuro-osteo-immune contexts. The presence of the periodontal ligament enables periodontitis to retain a certain degree of neural regulation and self-limiting capacity during early inflammation, whereas the inherent simplification of neural architecture in peri-implant tissues renders them more prone to shift toward a pro-inflammatory and pro-osteoclastic imbalance under biofilm and mechanical challenges.

The TRPV1-CGRP and SP-NK1R neuropeptide axes should not be simplistically categorized as purely “protective” or “destructive.” Rather, they perform markedly different biological functions depending on disease stage and microenvironmental conditions. Future studies are needed to delineate the dynamic alterations of neuropeptide profiles and their interaction nodes with classical osteoimmunological pathways, in order to develop more precise and stage-adapted neuromodulatory interventions for periodontitis and peri-implantitis.

## References

[1] Wang Y Wu Y Song J . Global and regional burden of periodontal disease in adults (1990-2021). Int Dent J. (2025) 75:103883. doi: 10.1016/j.identj.2025.103883. PMID: 40902507 PMC12444476

[2] Hu M Zhang R Wang R Wang Y Guo J . Global, regional, and national burden of periodontal diseases from 1990 to 2021 and predictions to 2040: an analysis of the global burden of disease study 2021. Front Oral Health. (2025) 6:1627746. doi: 10.3389/froh.2025.1627746. PMID: 40786185 PMC12332980

[3] Takahashi N Matsuda Y Sato K de Jong PR Bertin S Tabeta K . Neuronal TRPV1 activation regulates alveolar bone resorption by suppressing osteoclastogenesis via CGRP. Sci Rep. (2016) 6:29294. doi: 10.1038/srep29294. PMID: 27388773 PMC4937344

[4] Jiang Y Wang B . Sensory neuron TRPV1-mediated macrophage polarization and immune response regulate dental implant osseointegration. Tissue Cell. (2025) 99:103243. doi: 10.1016/j.tice.2025.103243. PMID: 41297248

[5] Diaz P Gonzalo E Villagra LJG Miegimolle B Suarez MJ . What is the prevalence of peri-implantitis? A systematic review and meta-analysis. BMC Oral Health. (2022) 22:449. doi: 10.1186/s12903-022-02493-8. PMID: 36261829 PMC9583568

[6] Scarano A Khater AGA Gehrke SA Serra P Francesco I Di Carmine M . Current status of peri-implant diseases: A clinical review for evidence-based decision making. J Funct Biomater. (2023) 14(4):210. doi: 10.3390/jfb14040210. PMID: 37103300 PMC10142594

[7] Kulalert W Wells AC Link VM Lim AI Bouladoux N Nagai M . The neuroimmune CGRP-RAMP1 axis tunes cutaneous adaptive immunity to the microbiota. Proc Natl Acad Sci USA. (2024) 121:e2322574121. doi: 10.1101/2023.12.26.573358. PMID: 38451947 PMC10945812

[8] Zhu Y Meerschaert KA Galvan-Pena S Bin NR Yang D Basu H . A chemogenetic screen reveals that Trpv1-expressing neurons control regulatory T cells in the gut. Science. (2024) 385:eadk1679. doi: 10.1126/science.adk1679. PMID: 39088603 PMC11416019

[9] Baral P Umans BD Li L Wallrapp A Bist M Kirschbaum T . Nociceptor sensory neurons suppress neutrophil and γδ T cell responses in bacterial lung infections and lethal pneumonia. Nat Med. (2018) 24:417–26. doi: 10.1038/nm.4501. PMID: 29505031 PMC6263165

[10] Chiu IM Heesters BA Ghasemlou N Von Hehn CA Zhao F Tran J . Bacteria activate sensory neurons that modulate pain and inflammation. Nature. (2013) 501:52–7. doi: 10.1038/nature12479. PMID: 23965627 PMC3773968

[11] Pinho-Ribeiro FA Verri WA Chiu IM . Nociceptor sensory neuron-immune interactions in pain and inflammation. Trends Immunol. (2017) 38:5–19. doi: 10.1016/j.it.2016.10.001. PMID: 27793571 PMC5205568

[12] Jiang Y Zhu Z Wang B Yuan Y Zhang Q Li Y . Neuronal TRPV1-CGRP axis regulates bone defect repair through Hippo signaling pathway. Cell Signal. (2023) 109:110779. doi: 10.1016/j.cellsig.2023.110779. PMID: 37336315

[13] Lu Y-Z Nayer B Singh SK Alshoubaki YK Yuan E Park AJ . CGRP sensory neurons promote tissue healing via neutrophils and macrophages. Nature. (2024) 628:604–11. doi: 10.1038/s41586-024-07237-y. PMID: 38538784 PMC11023938

[14] Chen J Sun W Zhu Y Zhao F Deng S Tian M . TRPV1: The key bridge in neuroimmune interactions. J Intensive Med. (2024) 4:442–52. doi: 10.1016/j.jointm.2024.01.008. PMID: 39310069 PMC11411435

[15] Zhu Z Jiang Y Li Z Du Y Chen Q Guo Q . Sensory neuron transient receptor potential vanilloid-1 channel regulates angiogenesis through CGRP *in vivo*. Front Bioeng Biotechnol. (2024) 12:1338504. doi: 10.3389/fbioe.2024.1338504. PMID: 38576442 PMC10991839

[16] Wang Q Qin H Deng J Xu H Liu S Weng J . Research progress in calcitonin gene-related peptide and bone repair. Biomolecules. (2023) 13:838. doi: 10.3390/biom13050838. PMID: 37238709 PMC10216440

[17] Lu Q Zheng Q Zhou Z Chen Y Chen Y Chen W . CGRP enhances the regeneration of bone defects by regulating bone marrow mesenchymal stem cells through promoting ANGPTL4 secretion by bone blood vessels. Adv Sci (Weinh). (2026) 13:e22295. doi: 10.1002/advs.202522295. PMID: 41487103 PMC12970192

[18] Zhao X Wu G Zhang J Yu Z Wang J . Activation of CGRP receptor-mediated signaling promotes tendon-bone healing. Sci Adv. (2024) 10:eadg7380. doi: 10.1126/sciadv.adg7380. PMID: 38457499 PMC10923525

[19] Wang Q Chen Y Ding H Cai Y Yuan X Lv J . Optogenetic activation of mechanical nociceptions to enhance implant osseointegration. Nat Commun. (2025) 16:3093. doi: 10.1038/s41467-025-58336-x. PMID: 40164597 PMC11958704

[20] Yuan Y Jiang Y Wang B Guo Y Gong P Xiang L . Deficiency of calcitonin gene-related peptide affects macrophage polarization in osseointegration. Front Physiol. (2020) 11:733. doi: 10.3389/fphys.2020.00733. PMID: 32848807 PMC7412000

[21] Qi X Yang G Xu Z Zhou M Liu T Du J . Neutrophil-initiated nociceptive ingrowth orchestrates inflammation resolution to potentiate bone regeneration. Bone Res. (2026) 14:9. doi: 10.1038/s41413-025-00481-6. PMID: 41554692 PMC12816638

[22] Deng L Gillis JE Chiu IM Kaplan DH . Sensory neurons: An integrated component of innate immunity. Immunity. (2024) 57:815–31. doi: 10.1016/j.immuni.2024.03.008. PMID: 38599172 PMC11555576

[23] Tynan A Tsaava T Gunasekaran M Bravo Iñiguez CE Brines M Chavan SS . TRPV1 nociceptors are required to optimize antigen-specific primary antibody responses to novel antigens. Bioelectron Med. (2024) 10:14. doi: 10.1186/s42234-024-00145-6. PMID: 38807193 PMC11134756

[24] Fang X Chen Y Ding H Huang C Hu H Zhang C . Staphylococcus aureus tames nociceptive neurons to suppress synovial macrophage responses for sustained infection in septic arthritis. Adv Sci (Weinh). (2025) 12:e2409251. doi: 10.1002/advs.202409251. PMID: 39960341 PMC11984863

[25] Pinho-Ribeiro FA Baddal B Haarsma R O’Seaghdha M Yang NJ Blake KJ . Blocking neuronal signaling to immune cells treats streptococcal invasive infection. Cell. (2018) 173:1083–1097.e22. doi: 10.1016/j.cell.2018.04.006. PMID: 29754819 PMC5959783

[26] Fang X Ding H Chen Y Wang Q Yuan X Zhang C . Wireless optogenetic targeting nociceptors helps host cells win the competitive colonization in implant-associated infections. Small Methods. (2024) 8:e2400216. doi: 10.1002/smtd.202400216. PMID: 39087367

[27] Miki K Takeshita N Yamashita M Kitamura M Murakami S . Calcitonin gene-related peptide regulates periodontal tissue regeneration. Sci Rep. (2024) 14:1344. doi: 10.1038/s41598-024-52029-z. PMID: 38228723 PMC10791604

[28] Siddiqui YD Nie X Wang S Abbasi Y Park L Fan X . Substance P aggravates ligature-induced periodontitis in mice. Front Immunol. (2023) 14:1099017. doi: 10.3389/fimmu.2023.1099017. PMID: 37122730 PMC10140340

[29] Wang L Zhao R Shi X Wei T Halloran BP Clark DJ . Substance P stimulates bone marrow stromal cell osteogenic activity, osteoclast differentiation, and resorption activity *in vitro*. Bone. (2009) 45:309–20. doi: 10.1016/j.bone.2009.04.203. PMID: 19379851 PMC2706279

[30] Niedermair T Schirner S Seebröker R Straub RH Grässel S . Substance P modulates bone remodeling properties of murine osteoblasts and osteoclasts. Sci Rep. (2018) 8:9199. doi: 10.1038/s41598-018-27432-y. PMID: 29907830 PMC6003941

[31] Hofman M Rabenschlag F Andruszkow H Andruszkow J Möckel D Lammers T . Effect of neurokinin-1-receptor blockage on fracture healing in rats. Sci Rep. (2019) 9:9744. doi: 10.1038/s41598-019-46278-6. PMID: 31278316 PMC6611911

[32] Johnson MB Suptela SR Sipprell SE Marriott I . Substance P exacerbates the inflammatory and pro-osteoclastogenic responses of murine osteoclasts and osteoblasts to Staphylococcus aureus. Inflammation. (2023) 46:256–69. doi: 10.1007/s10753-022-01731-z. PMID: 36040535 PMC10314328

[33] Li F-X-Z Xu F Lin X Wu F Zhong J-Y Wang Y . The role of substance P in the regulation of bone and cartilage metabolic activity. Front Endocrinol (Lausanne). (2020) 11:77. doi: 10.3389/fendo.2020.00077. PMID: 32180759 PMC7059306

[34] Sipprell SE Krueger QA Mills EL Marriott I Johnson MB . Substance P augments chemokine production by Staphylococcus aureus infected murine osteoclasts. Inflammation. (2025) 48:1–13. doi: 10.1007/s10753-025-02280-x. PMID: 40056352 PMC12353046

[35] Schank JR Heilig M . Substance P and the neurokinin-1 receptor: the new CRF. Int Rev Neurobiol. (2017) 136:151–75. doi: 10.1016/bs.irn.2017.06.008. PMID: 29056150

[36] Goto T Kido MA Yamaza T Tanaka T . Substance P and substance P receptors in bone and gingival tissues. Med Electron Microsc. (2001) 34:77–85. doi: 10.1007/s007950170001. PMID: 11685656

[37] Fristad I Vandevska-Radunovic V Fjeld K Wimalawansa S Hals Kvinnsland I . NK1, NK2, NK3 and CGRP1 receptors identified in rat oral soft tissues, and in bone and dental hard tissue cells. Cell Tissue Res. (2003) 311:383–91. doi: 10.1007/s00441-002-0691-z. PMID: 12658446

[38] Azuma H Kido J Ikedo D Kataoka M Nagata T . Substance P enhances the inhibition of osteoblastic cell differentiation induced by lipopolysaccharide from Porphyromonas gingivalis. J Periodontol. (2004) 75:974–81. doi: 10.1902/jop.2004.75.7.974. PMID: 15341355

[39] Winning L El Karim IA Linden GJ Irwin CR Killough SA Lundy FT . Differential regulation of NPY and SP receptor expression in STRO-1+ve PDLSCs by inflammatory cytokines. J Periodontal Res. (2022) 57:186–94. doi: 10.1111/jre.12952. PMID: 34773642

[40] Yan K Lin Q Tang K Liu S Du Y Yu X . Substance P participates in periodontitis by upregulating HIF-1α and RANKL/OPG ratio. BMC Oral Health. (2020) 20:27. doi: 10.1186/s12903-020-1017-9. PMID: 32000757 PMC6993464

[41] Yu XJ Xiao CJ Du YM Liu S Du Y Li S . Effect of hypoxia on the expression of RANKL/OPG in human periodontal ligament cells *in vitro*. Int J Clin Exp Pathol. (2015) 8:12929–35. PMC4680431 26722486

[42] Yamaza T Kido MA Wang B Danjo A Shimohira D Murata N . Distribution of substance P and neurokinin-1 receptors in the peri-implant epithelium around titanium dental implants in rats. Cell Tissue Res. (2009) 335:407–15. doi: 10.1007/s00441-008-0720-7. PMID: 19015883

[43] Li J Cui Z Gong H Zhang Y Yuan Z Zhang Z . Sleep deficiency exacerbates periodontal inflammation via trigeminal TRPV1 neurons. Proc Natl Acad Sci USA. (2025) 122:e2424169122. doi: 10.1016/j.identj.2025.104045. PMID: 40489620 PMC12184432

[44] Wang L Hou S Sabsovich I Guo T-Z Wei T Kingery WS . Mice lacking substance P have normal bone modeling but diminished bone formation, increased resorption, and accelerated osteopenia with aging. Bone. (2021) 144:115806. doi: 10.1016/j.bone.2020.115806. PMID: 33333245 PMC7856000

[45] Kvinnsland I Kvinnsland S . Changes in CGRP-immunoreactive nerve fibres during experimental tooth movement in rats. Eur J Orthod. (1990) 12:320–9. doi: 10.1093/ejo/12.3.320. PMID: 2205509

[46] Nagayama T Seiryu M Deguchi T Kano M Suzuki T Takano-Yamamoto T . Increase of CGRP-containing nerve fibers in the rat periodontal ligament after luxation. Cell Mol Neurobiol. (2012) 32:391–7. doi: 10.1007/s10571-011-9767-1. PMID: 22038237 PMC11498513

[47] Nicolay OF Davidovitch Z Shanfeld JL Alley K . Substance P immunoreactivity in periodontal tissues during orthodontic tooth movement. Bone Miner. (1990) 11:19–29. doi: 10.1016/0169-6009(90)90012-5. PMID: 1702686

[48] Trulsson M . Sensory and motor function of teeth and dental implants: a basis for osseoperception. Clin Exp Pharmacol Physiol. (2005) 32:119–22. doi: 10.1111/j.1440-1681.2005.04139.x. PMID: 15730446

[49] Huang Y Jacobs R Van Dessel J Bornstein MM Lambrichts I Politis C . A systematic review on the innervation of peri‐implant tissues with special emphasis on the influence of implant placement and loading protocols. Clin Oral Implants Res. (2015) 26:737–46. doi: 10.1111/clr.12344. PMID: 24502689

[50] Ivanovski S Bartold PM Huang YS . The role of foreign body response in peri‐implantitis: What is the evidence? Periodontol 2000. (2022) 90:176–85. doi: 10.1111/prd.12456. PMID: 35916872 PMC9804527

[51] Kheder W Bouzid A Venkatachalam T Talaat IM Elemam NM Raju TK . Titanium particles modulate lymphocyte and macrophage polarization in peri-implant gingival tissues. Int J Mol Sci. (2023) 24:11644. doi: 10.3390/ijms241411644. PMID: 37511404 PMC10381089

[52] Toledano-Serrabona J Bosch BM Díez-Tercero L Gil FJ Camps-Font O Valmaseda-Castellón E . Evaluation of the inflammatory and osteogenic response induced by titanium particles released during implantoplasty of dental implants. Sci Rep. (2022) 12:15790. doi: 10.1038/s41598-022-20100-2. PMID: 36138061 PMC9500064

[53] Fort BP Dubyak GR Greenfield EM . Lysosomal disruption by orthopedic wear particles induces activation of the NLRP3 inflammasome and macrophage cell death by distinct mechanisms. J Orthop Res. (2021) 39:493–505. doi: 10.1002/jor.24826. PMID: 32779803 PMC8201664

[54] Yazdi AS Guarda G Riteau N Drexler SK Tardivel A Couillin I . Nanoparticles activate the NLR pyrin domain containing 3 (Nlrp3) inflammasome and cause pulmonary inflammation through release of IL-1α and IL-1β. Proc Natl Acad Sci USA. (2010) 107:19449–54. doi: 10.1073/pnas.1008155107. PMID: 20974980 PMC2984140

[55] Kim B-G Lee P-H Lee S-H Park M-K Jang A-S . Effect of TiO2 nanoparticles on inflammasome-mediated airway inflammation and responsiveness. Allergy Asthma Immunol Res. (2017) 9:257–64. doi: 10.4168/aair.2017.9.3.257. PMID: 28293932 PMC5352577

[56] Eger M Hiram-Bab S Liron T Sterer N Carmi Y Kohavi D . Mechanism and prevention of titanium particle-induced inflammation and osteolysis. Front Immunol. (2018) 9:2963. doi: 10.3389/fimmu.2018.02963. PMID: 30619321 PMC6305459

[57] Wei X Zhang X Zuscik MJ Drissi MH Schwarz EM O'Keefe RJ . Fibroblasts express RANKL and support osteoclastogenesis in a COX-2-dependent manner after stimulation with titanium particles. J Bone Miner Res. (2005) 20:1136–48. doi: 10.1359/jbmr.050206. PMID: 15940366

[58] Eger M Sterer N Liron T Kohavi D Gabet Y . Scaling of titanium implants entrains inflammation-induced osteolysis. Sci Rep. (2017) 7:39612. doi: 10.1038/srep39612. PMID: 28059080 PMC5216395

[59] Dodo CG Meirelles L Aviles-Reyes A Ruiz KGS Abranches J Cury AADB . Pro-inflammatory analysis of macrophages in contact with titanium particles and porphyromonas gingivalis. Braz Dent J. (2017) 28:428–34. doi: 10.1590/0103-6440201701382. PMID: 29160393 PMC6982405

[60] Hensellek S Brell P Schaible HG Bräuer R Segond von Banchet G . The cytokine TNFalpha increases the proportion of DRG neurones expressing the TRPV1 receptor via the TNFR1 receptor and ERK activation. Mol Cell Neurosci. (2007) 36:381–91. doi: 10.1016/j.mcn.2007.07.010. PMID: 17851089

[61] Meng J Wang J Steinhoff M Dolly JO . TNFα induces co-trafficking of TRPV1/TRPA1 in VAMP1-containing vesicles to the plasmalemma via Munc18-1/syntaxin1/SNAP-25 mediated fusion. Sci Rep. (2016) 6:21226. doi: 10.1038/srep21226. PMID: 26888187 PMC4758037

[62] Inoue A Ikoma K Morioka N Kumagai K Hashimoto T Hide I . Interleukin-1beta induces substance P release from primary afferent neurons through the cyclooxygenase-2 system. J Neurochem. (1999) 73:2206–13. doi: 10.1016/s0021-5198(19)47611-7 10537081

[63] Pettersson M Kelk P Belibasakis GN Bylund D Molin Thorén M Johansson A . Titanium ions form particles that activate and execute interleukin-1β release from lipopolysaccharide-primed macrophages. J Periodontal Res. (2017) 52:21–32. doi: 10.1111/jre.12364. PMID: 26987886 PMC5297875

[64] Hamlet SM Lee RSB Moon HJ Alfarsi MA Ivanovski S . Hydrophilic titanium surface-induced macrophage modulation promotes pro-osteogenic signalling. Clin Oral Implants Res. (2019) 30:1085–96. doi: 10.1111/clr.13522. PMID: 31397920

[65] Alfarsi MA Hamlet SM Ivanovski S . Titanium surface hydrophilicity modulates the human macrophage inflammatory cytokine response. J BioMed Mater Res A. (2014) 102:60–7. doi: 10.1002/jbm.a.34666. PMID: 23595995

[66] Hotchkiss KM Reddy GB Hyzy SL Schwartz Z Boyan BD Olivares-Navarrete R . Titanium surface characteristics, including topography and wettability, alter macrophage activation. Acta Biomater. (2016) 31:425–34. doi: 10.1016/j.actbio.2015.12.003. PMID: 26675126 PMC4728000

[67] Ma Q-L Zhao L-Z Liu R-R Jin B-Q Song W Wang Y . Improved implant osseointegration of a nanostructured titanium surface via mediation of macrophage polarization. Biomaterials. (2014) 35:9853–67. doi: 10.1016/j.biomaterials.2014.08.025. PMID: 25201737

[68] Lee RSB Hamlet SM Moon H-J Ivanovski S . Re-establishment of macrophage homeostasis by titanium surface modification in type II diabetes promotes osseous healing. Biomaterials. (2021) 267:120464. doi: 10.1016/j.biomaterials.2020.120464. PMID: 33130322

[69] Abaricia JO Shah AH Ruzga MN Olivares-Navarrete R . Surface characteristics on commercial dental implants differentially activate macrophages *in vitro* and *in vivo*. Clin Oral Implants Res. (2021) 32:487–97. doi: 10.1111/clr.13717. PMID: 33502059 PMC8207525

[70] Degasperi W Andersson P Verrocchi D Sennerby L . One-year clinical and radiographic results with a novel hydrophilic titanium dental implant. Clin Implant Dent Relat Res. (2014) 16:511–19. doi: 10.1111/cid.12022. PMID: 23190341

[71] Ferreira ACRDM Nogueira TE de Oliveira BS Dias AP Leles JLR de Souza PPC . Changes in stability and marginal bone level around implants with hydrophilic and hydrophobic surfaces for posterior tooth replacement: A 1-year randomized clinical trial. J Dent. (2025) 157:105696. doi: 10.1016/j.jdent.2025.105696. PMID: 40101852

[72] Huang Y Jacobs R Van Dessel J Bornstein MM Lambrichts I Politis C . A systematic review on the innervation of peri-implant tissues with special emphasis on the influence of implant placement and loading protocols. Clin Oral Implants Res. (2015) 26:737–46. doi: 10.1111/clr.12344. PMID: 24502689

